# Quercetin increases the antioxidant capacity of the ovary in menopausal rats and in ovarian granulosa cell culture in vitro

**DOI:** 10.1186/s13048-018-0421-0

**Published:** 2018-06-21

**Authors:** Jiao Wang, Xin Qian, Qiang Gao, Chunmei Lv, Jie Xu, Hongbo Jin, Hui Zhu

**Affiliations:** Department of Physiology, College of Basic Medical Sciences, Harbin Medical University, Harbin, China

**Keywords:** Ovary, Menopause, Oxidative stress, Quercetin

## Abstract

**Background:**

Menopause is the most important sign of aging in women, and the ovary is the organ most sensitive to aging. Quercetin is a potential antioxidant and free radical scavenger that is widely found in fruits, vegetables, and leaves. However, the effect of quercetin on ovarian aging has not been elucidated, and the mechanism underlying its antioxidative effect remains unclear. The purpose of the current study was to investigate whether quercetin protects ovarian function by decreasing oxidative stress.

**Methods:**

In an in vivo experiment, female menopausal rats (12 months old) were intragastrically administered quercetin at three doses (12.5 mg/kg, 25 mg/kg, and 50 mg/kg) for 90 days, and the estrous cycles were determined by vaginal smearing. In an in vitro experiment, rat primary ovarian granulosa cells were cultured and treated with H_2_O_2_ (400 μM) alone or H_2_O_2_ plus quercetin at 5 μM, 20 μM, or 50 μM. The levels of the hormones estradiol (E_2_), progesterone (P), follicle-stimulating hormone (FSH) and luteinizing hormone (LH) were detected by radioimmunoassay. The serum levels of total antioxidant capacity (T-AOC), superoxide dismutase (SOD), glutathione (GSH), glutathione peroxidase (GSH-PX) and glutathione S-transferase (GST) were examined. The expression levels of the oxidative stress-related genes SOD-1, catalase (CAT) and glutathione synthetase (GSS) in the ovaries and ovarian granulosa cells were detected by Western blot.

**Results:**

The in vivo results demonstrated that quercetin had no effects on ovarian morphology, hormone secretion, or the estrous cycle in menopausal rats. Although no significant changes were detected in the serum levels of T-AOC, SOD, GSH, GSH-PX, and GST between the quercetin and control groups, the mRNA and protein expression levels of the oxidative stress-related genes SOD-1, CAT and GSS in menopausal rat ovaries were increased by low-dose quercetin. Moreover, the in vitro results demonstrated that quercetin significantly rescued the decrease in cell viability by H_2_О_2_-induced oxidative stress and enhanced the H_2_O_2_-induced decrease in expression of oxidative stress-related proteins.

**Conclusions:**

Together, the results of this study indicated that quercetin increased the antioxidant capacity of the ovary by upregulating the expression of some oxidative stress-related genes both in vivo and in vitro.

## Background

Aging is an inevitable physiological process in the body, characterized by a time-dependent decline in physiological functions of the major systems. Oxidative stress is one of the most important reasons for aging because of the accumulation of daily metabolic wastes [1, 2]. Oxidative stress reactions are induced when the generation of free radicals, including reactive oxygen species (ROS), exceeds the scavenging ability of the body. Decreased activity of oxidative stress-related enzymes, including superoxide dismutase (SOD), catalase (CAT), glutathione (GSH), and glutathione-S-transferase (GST), has been found in various tissues in animal models of aging [1].

Quercetin, a bioflavonoid widely found in many fruits, vegetables, and leaves [3], is well known for its pharmacological effects against various diseases, such as cancer, inflammation, thrombosis and hypertension [4–7]. In some pathological conditions, quercetin has a strong scavenging effect on free radical production by increasing the activities of GSH, SOD, CAT, GSH-PX and glutathione reductase [8, 9].

Ovary is the organ that is the most sensitive to aging. The aging of the ovary is primarily characterized by decreases in the number of follicles and in oocyte quality [10]. Menopause is the most important sign of aging in women. After menopause, with the decline in ovarian function, the antioxidant capacity of the ovary may be reduced. Studies show that excessive ovarian ROS production in menopausal women can damage the normal structure and function of cells by disrupting the lipid, fatty acid and protein composition of cells [10]. Okatani et al. reported that the SOD and GSH-PX enzyme activities in the ovaries of postmenopausal women are significantly lower than those in premenopausal women [11].

Recently, some studies have reported the role of quercetin on ovarian functions in different animal models. Naseer et al. found that quercetin supplementation significantly improves the follicular development, minimize granulosa cells apoptosis in heat stress rabbits [12]. Another study, by Victor et al., found that quercetin treatment exerts preventive effects on cadmium chloride (CdCl_2_) - induced toxicity in the uterus and ovaries of Wistar rats by its antioxidant and anti-apoptotic actions [13]. Some in vitro studies also found the possible role of quercetin on ovarian cells. Two groups have reported that quercetin contributes potentially to prevent T-2 toxin or cadmium induced oxidative damage and apoptosis in cultured granulosa cells from porcine or chicken ovarian follicles [14, 15]. Although Chen et al. found that quercetin has positive effects by affecting the ovarian follicular reserve in aging rats [16], the effect mechanism of quercetin on ovarian aging has not been elucidated.

In this study, female menopausal rats and primary cultured ovarian granulosa cells were used to investigate whether quercetin could protect ovarian function and whether the mechanism of action was related to its antioxidant effects.

## Methods

### Animals and experimental groups

Menopausal female Sprague-Dawley (SD) rats (12 months old and weighing 348 ± 40.05 g) were obtained from the animal experiment center of Harbin Medical University. The Institutional Animal Care and Use Committee (IACUC) of Harbin Medical University approved all experiments. Twenty-eight rats were randomly divided into 4 groups of 7 rats each: control (1% methylcellulose dissolved in normal saline), low-dose quercetin (QL, 12.5 mg/kg), middle-dose quercetin (QM, 25 mg/kg) and high-dose quercetin (QH, 50 mg/kg). Control solution and quercetin (dissolved in control solution) were administered intragastrically to rats for 90 days.

### Estrous cycle determination by vaginal smearing

To assess the stages of the estrous cycle in the rats, vaginal smears were examined daily for 15 days before and after treatments. The vaginal smears were stained using 0.04% trypan blue, and the cytological characteristics were examined using bright-field microscopy.

### Primary ovarian granulosa cell culture

Immature female SD rats (21 days old) were subcutaneously injected with pregnant mare serum gonadotropin (PMSG)50 IU and were sacrificed 48 h later. Removed ovaries were immediately washed with phosphate-buffered saline (PBS) and placed in DMEM/F12 medium. Granulosa cells were harvested in the medium by needle puncture of ovarian follicles under a dissecting microscope and then purified by filtration with a 200-μm stainless steel mesh. After the centrifugation at 1000×g for 5 min, the cells were resuspended in medium and counted in a hemocytometer. The cells were seeded in 96-well plates (1 × 10^5^ cells/well) or in 6-well plates (1 × 10^7^ cells/well) and cultured in DMEM/F12 medium supplemented with 15% FBS, testosterone (10^− 7^ M), 100 U/mL penicillin and 100 mg/mL streptomycin at 37 °C and 5% CO_2_ for 48 h to allow the cells to attach. The cells were randomly divided into five groups – control, H_2_O_2_ (400 μM), and H_2_O_2_ plus three concentrations of quercetin (5 μM, 20 μM, and 50 μM) – and cultured for 6 h. At the end of the experiment, estradiol (E_2_) production in the culture medium was determined by radioimmunoassay, and total cellular proteins were extracted and used to determine protein expression by Western blot.

### Cell viability determination

Cellular viability was measured via the CCK-8 assay. At the end of culture, cells in 96-well plates were incubated in 200 μL DMEM/F12 supplemented with 20 μL of CCK-8 reagent for 3 h at 37 °C in a 5% CO_2_ incubator. The optical density (OD) value of each well was measured at a wavelength of 450 nm using a microplate reader. The relative cellular viability = the OD value of the test group well / the mean OD value of the control group. Each group was established in five wells, and each measurement was repeated at least 2 times.

### Hormone detection by radioimmunoassay

The levels of estradiol (E_2_), progesterone (P), follicle stimulating hormone (FSH) and luteinizing hormone (LH) were detected using commercial radioimmunoassay kits (Sino-UK Institute of Biological Technology, Beijing, China).

### Measurement of antioxidant indices

The serum activities of antioxidant indices (T-AOC, SOD, GSH, GSH-PX, and GST) were measured using commercial biochemical kits (Sino-UK Institute of Biological Technology, Beijing, China) according to the manufacturer’s instructions.

### Quantitative real-time PCR

The mRNA expression of SOD-1, CAT and GSS in the ovaries was assessed using real-time PCR. Briefly, total RNA was extracted from ovaries using TRIzol reagent (Invitrogen, Carlsbad, CA, USA). First-strand cDNA was synthesized from 2 μg of total RNA using a PrimeScript 1st Strand cDNA Synthesis Kit (Takara Bio Inc., Dalian, China) following the supplier’s instructions. Real-time PCR was performed in 20 μL mixtures using an SYBR Premix Ex Taq™ II Kit (Takara Bio Inc., Dalian, China) and containing 1 μL of cDNA template, 0.5 μM forward and reverse primers (for characteristics of primers and real-time RT-PCR conditions, please see Table 1), and 10 μL of SYBR Premix Ex Taq™ II. The mRNA expression levels of genes were normalized to the level of β-actin mRNA expression. The data were evaluated by the ΔΔCt method using an Applied Biosystems 7500 Real-Time PCR System.

**Table 1:** Characteristics of Primers and Real-Time PCR Conditions

Gene	Forward primer (5′ → 3′)	Reverse primer (5′ → 3′)	Accession number
SOD-1	AGGGCGTCATTCACTTCGAG	CCTCTCTTCATCCGCTGGAC	NM_017050.1
CAT	TTTTCACCGACGAGATGGCA	CTGACTCTCCAGCGACTGTG	NM_012520.2
GSS	GAGTTTGAGCTTGGCGAGCAG	ATGGGGCATACGTCACCAC	NM_012962.1
β-actin	CACCCGCGAGTACAACCTTC	CCCATACCCACCATCACACC	NM_031144

Two-step real-time PCR conditions: Stage 1: Initial denaturation = 30 s at 95 °C; Stage 2: PCR amplification, 5 s at 95 °C and 34 s at 60 °C; extension = 30 s at 72 °C

### Western blot analysis

The protein expression levels of SOD-1, CAT and GSS were assessed via Western blot analysis. Briefly, total protein (20 μg) was separated by 10% SDS-PAGE and transferred to a polyvinylidene difluoride (PVDF) membrane with a Trans-Blot SD semidry transfer cell (Bio-Rad Laboratories, Richmond, Calif.). The membranes were blocked with 5% skim milk powder dissolved in Tris-buffered saline containing 0.1% Tween 20. The membranes were then incubated with SOD-1/CAT/GSS rabbit polyclonal antibodies (Santa Cruz Biotechnology, Santa Cruz, CA, USA) overnight at 4 °C and then with a horseradish peroxidase (HRP)-conjugated goat anti-rabbit IgG antibody (Santa Cruz Biotechnology, Santa Cruz, CA, USA) for 1 h at room temperature. The protein bands were visualized using Pierce ECL Western Blotting Substrate (Engreen Biosystem, Beijing, China). The relative density of bands was assessed by densitometry using ImageJ software (http://rsbweb.nih.gov/ij/download.html).

### Hematoxylin and eosin (HE) staining

The ovaries were isolated and fixed in 4% buffered formaldehyde in 0.1 M phosphate buffer for 48 h and then were processed for paraffin embedding and sectioning. Serial sections of 5 μm thickness were cut with a Leica RM 2016 rotator microtome. The sections were dewaxed with xylene, rehydrated with graded concentrations of ethanol and then stained with hematoxylin and eosin and evaluated via light microscopy.

### Immunohistochemistry

Paraffin sections of rat ovary were dewaxed with xylene and rehydrated by graded concentrations of ethanol. After gradual hydration, the slides were incubated in citrate buffer (pH 6.0) at 95 °C for 20 min and then cooled for 1 h at room temperature. The sections were treated with 0.3% H_2_O_2_ for 10 min in a dark room to inhibit endogenous peroxidase activity. After blocking nonspecific reactions for 30 min, the sections were then incubated with SOD-1/CAT/GSS rabbit polyclonal antibody (Santa Cruz Biotechnology, Santa Cruz, CA, USA) at 4 °C for 12 h. Then, the slides were incubated with the second antibody at 37 °C for 1 h and counterstained with DAB and hematoxylin.

### Statistical analyses

The data are expressed as the mean ± standard deviation. Differences among the means were evaluated by one-way ANOVA using the SPSS 13.0 statistical software package (SPSS, Inc., Chicago, IL, USA). *P* < 0.05 was considered to indicate a statistically significant difference.

## Results

### Effect of quercetin on the estrous cycle in menopausal rats

The estrous cycle of menopausal rats was determined by daily vaginal cytology for 15 days before and after administration of quercetin. A normal estrous cycle in a female rat lasts for 4 to 5 days and can be divided into four time periods: proestrus, estrus, metestrus and diestrus. Before administration of quercetin, 93.3% of rats (28/30) had a long estrous cycle (6.4 ± 1.0 days), and each period was irregular or prolonged to varying degrees (Table 2), indicating that most of the rats (12 months old) had lost normal estrous cycle characteristics and entered menopause. The rats (7 rats/group) were intragastrically treated with control solution or one of three doses of quercetin (12.5 mg/kg, 25 mg/kg, and 50 mg/kg) for 90 days. After quercetin administration, estrous cycles of all rats (15 months old) stopped at either estrus (46.43%) or diestrus (53.57%) (Table 3), indicating that the estrous cycle had been terminated in all rats [17]. Furthermore, no obvious differences in the estrous cycle were observed between the control group and the groups treated with the three doses of quercetin, indicating that quercetin did not affect the estrous cycle of menopausal rats.

**Table 2:** The estrous cycle in menopausal rats before administration of quercetin

Estrous cycle	No. of rats	Percentage
Proestrus lasts for more than 2 days	4	13.33%
Estrus lasts for more than 2 days	15	50.00%
Metestrus lasts for more than 2 days	22	73.33%
Diestrus s lasts for more than 3 days	7	23.33%

**Table 3:** The estrous cycle in menopausal rats after administration of quercetin

Estrous cycle	Control	QL	QM	QH	Total	Percentage
Continuous estrus	2	4	4	3	13	46.43%
Continuous diestrus	5	3	3	4	15	53.57%
Total	7	7	7	7	28	100%

### Effect of quercetin on ovarian weight and morphologic change in menopausal rats

To test the effect of quercetin on ovarian weight in menopausal rats, the ovaries were collected after 90 days of quercetin administration and then weighed. The average weights of ovaries were 0.104 ± 0.040 g (control group), 0.089 ± 0.020 g (low-dose quercetin), 0.087 ± 0.033 g (middle-dose quercetin), and 0.105 ± 0.048 g (high-dose quercetin); no significant differences between control and quercetin groups were observed (*P* > 0.05). Based on these results, quercetin had no effect on ovary weight in menopausal rats.

**Fig. 1:**
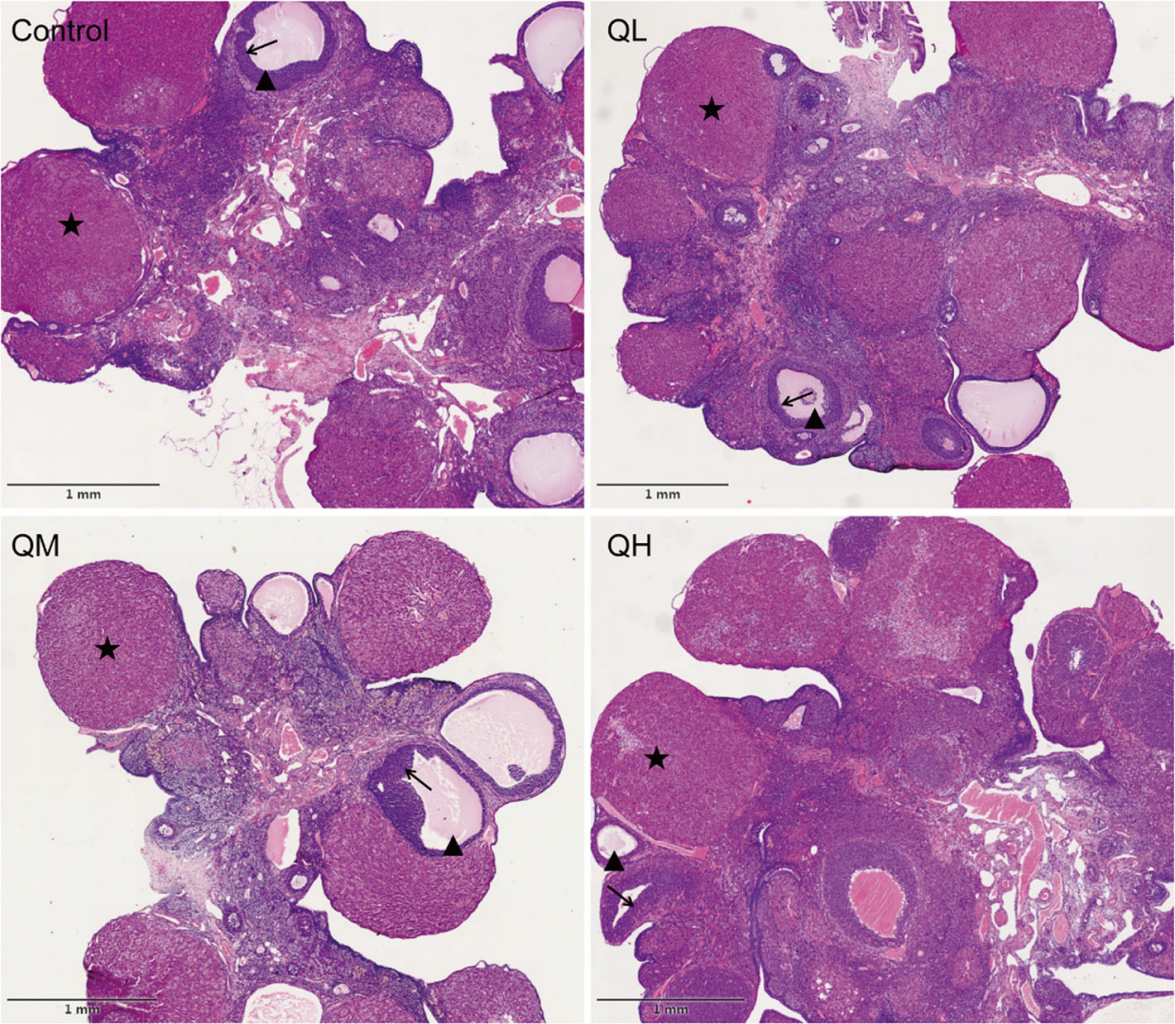
Effect of quercetin on morphology of menopausal rats. Rats were treated with different concentrations of quercetin or with the vehicle (1% methylcellulose) for 3 months. After administration, the ovary was sectioned and stained with hematoxylin and eosin. Scale bars, 1 mm. Quercetin had no obvious influence on ovarian morphology. ★corpus luteum;▲growing follicles;→granulosa cell

To further test the effect of quercetin on morphological changes to ovaries in menopausal rats, paraffin sections of ovaries were stained with hematoxylin and eosin (Fig. 1). The ovaries of menopausal rats demonstrated obvious characteristics of aging in both quercetin and control groups, including the exhaustion of the resting follicle reserve and a marked increase in the proportion of ovarian stroma.

### Effect of quercetin on serum hormone levels in menopausal rats

**Fig. 2:**
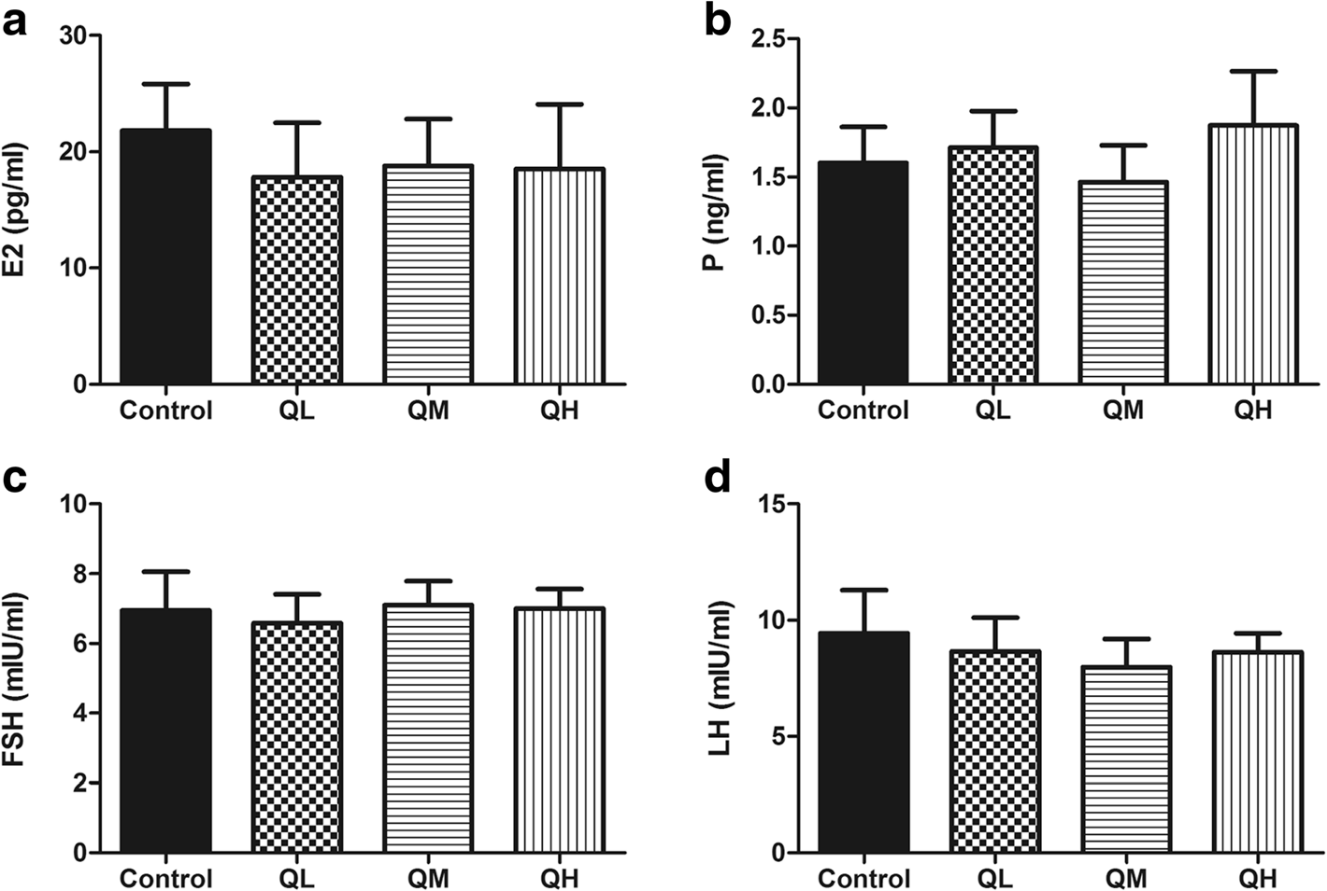
Effect of quercetin on serum hormone levels of menopausal rats. The serum levels of estradiol (E_2_), progesterone (P), follicle-stimulating hormone (FSH) and luteinizing hormone (LH) were detected using commercial radioimmunoassay kits. Data are presented as the *Mean* ± *SD* (*n* = 7). **a** E_2_ levels in menopausal rats; **b** P levels in menopausal rats; **c** FSH levels in menopausal rats; **d** LH levels in menopausal rats. Compared with the control group, the serum hormone levels in quercetin groups did not change significantly. (quercetin vs control, *P*>0.05)

The serum levels of the hormones E_2_, P, FSH and LH in menopausal rats were detected by radioimmunoassay, which showed that serum levels of E_2_, P, FSH and LH in the control group were 21.8 ± 1.7 (pg/ml), 1.603 ± 0.261 (ng/ml), 6.95 ± 0.42 (mIU/ml), and 9.45 ± 0.69 (mIU/ml), respectively. Compared with these levels in the control group, the serum levels of these four hormones did not change significantly after treatment with any of the three doses of quercetin (*P* > 0.05, Fig. 2). Additionally, no significant differences were detected among the groups treated with the three doses of quercetin, indicating that quercetin had no effects on the serum hormone levels in menopausal rats.

### Effect of quercetin on serum antioxidant factor levels in menopausal rats

**Fig. 3:**
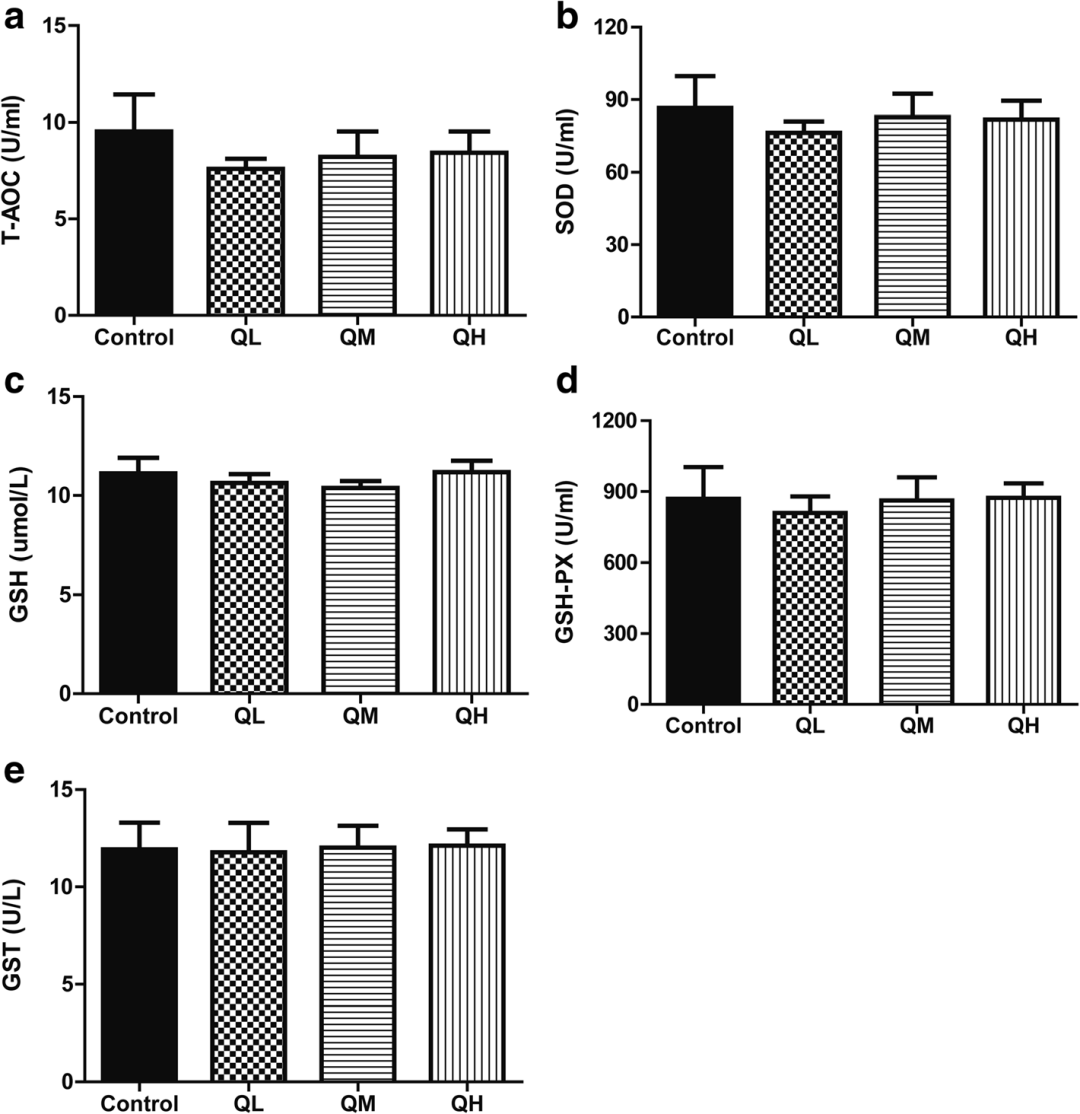
Effect of quercetin on the serum levels of T-AOC, SOD, GSH, GSH-PX, and GSH-ST of menopausal rats. The serum activities of antioxidant indices were measured by radioimmunoassay. Data are presented as the *Mean* ± *SD*. **a** total antioxidant capacity (T-AOC); **b** superoxide dismutase (SOD); **c** glutathione (GSH); **d** glutathione peroxidase (GSH-PX); **e** glutathione-S-transferase (GST). Compared with the control group, the antioxidant indices in quercetin groups did not change significantly. (quercetin vs control, *P* > 0.05)

T-AOC, SOD, GSH, GSH-PX and GST are the most important antioxidant indices in the body. To determine whether quercetin played a protective role by affecting antioxidant production in menopausal rats, the serum levels of T-AOC, SOD, GSH, GSH-PX and GST in the rats were examined. No significant changes in serum levels of the five antioxidant indices were observed between the three quercetin groups and the control group (*P* > 0.05, Fig. 3), indicating that quercetin had no effects on the serum levels of antioxidant factors in menopausal rats.

### Effect of quercetin on expression of SOD-1, CAT and GSS in the ovary of menopausal rats

**Fig. 4:**
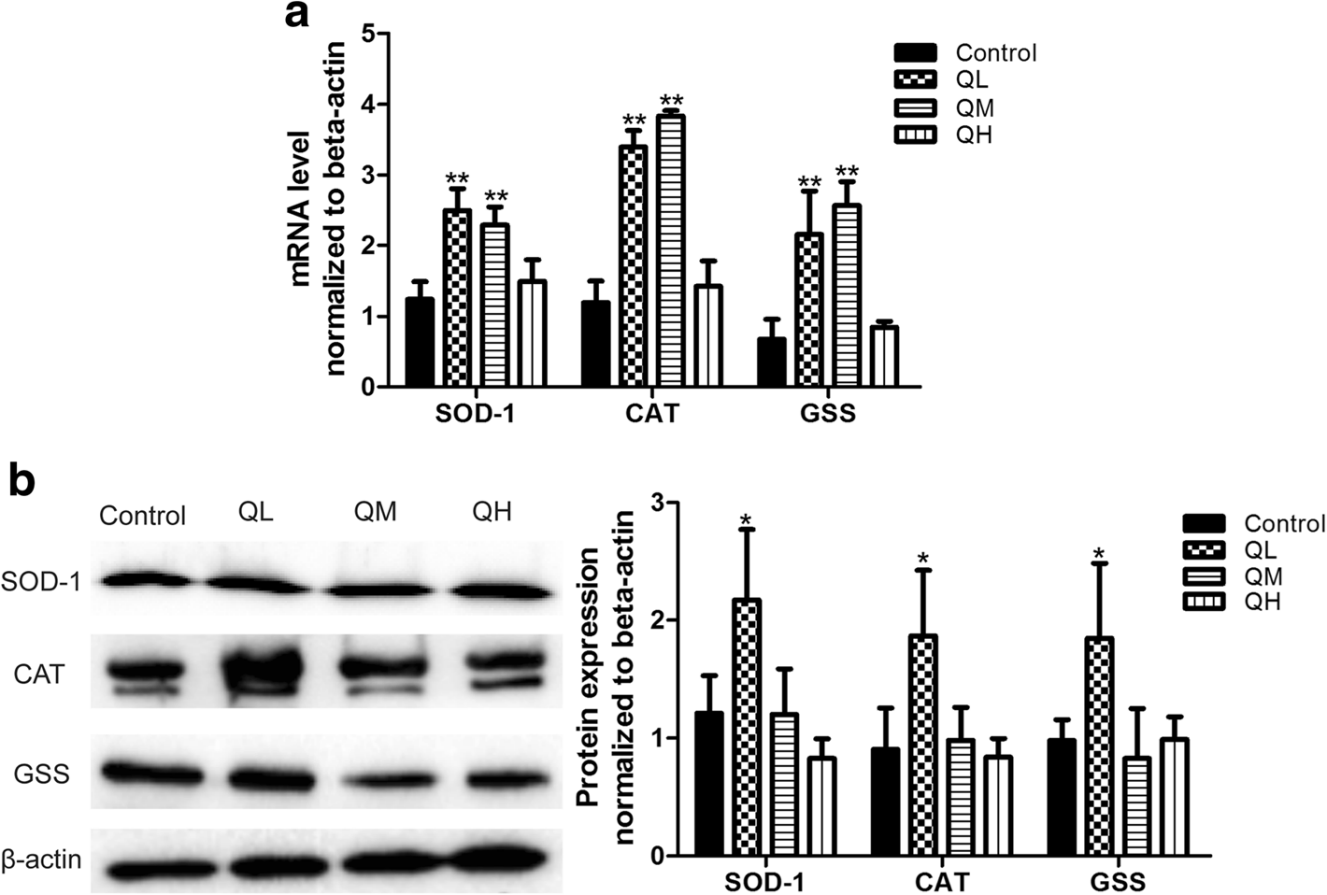
The expression of SOD-1, CAT and GSS in the ovary of menopausal rats. **a** The mRNA expression levels of genes as detected by real-time PCR. Data are presented as the *Mean* ± *SD* of four independent determinations. The mRNA expression levels of the three genes in the low-dose and middle-dose quercetin groups were higher than those in the control group. **b** Protein expression of genes as detected by Western blot. Data are expressed as the *Mean* ± *SD*. The protein expression levels of the three genes in the low-dose quercetin group were higher than those in the control group. (quercetin vs control, ^*^P < 0.05, ^**^P < 0.01)

To further determine whether quercetin affected the expression of oxidative stress-related genes in menopausal rats, the expression of mRNA and protein of SOD-1, CAT and GSS in ovaries was detected using real-time PCR and Western blot. The results are presented in Fig. 4. The mRNA expression levels of SOD-1, CAT and GSS in the low-dose and middle-dose quercetin groups were higher than those in the control group (*P* < 0.01, Fig. 4a). However, no significant difference in mRNA expression of SOD-1, CAT and GSS was detected between the high-dose quercetin group and the control group (*P* > 0.05). These results indicated that quercetin used within the range of doses in this study could increase the mRNA expression of SOD-1, CAT and GSS in menopausal rat ovaries. Western blot results showed that only rats treated with the low dose of quercetin demonstrated increased protein expression of SOD-1, CAT and GSS compared with that in control rats (*P* < 0.05). Middle-dose and high-dose quercetin treatments had no effects on the expression of these proteins in the menopausal rat ovary (P > 0.05; Fig. 4b).

**Fig. 5:**
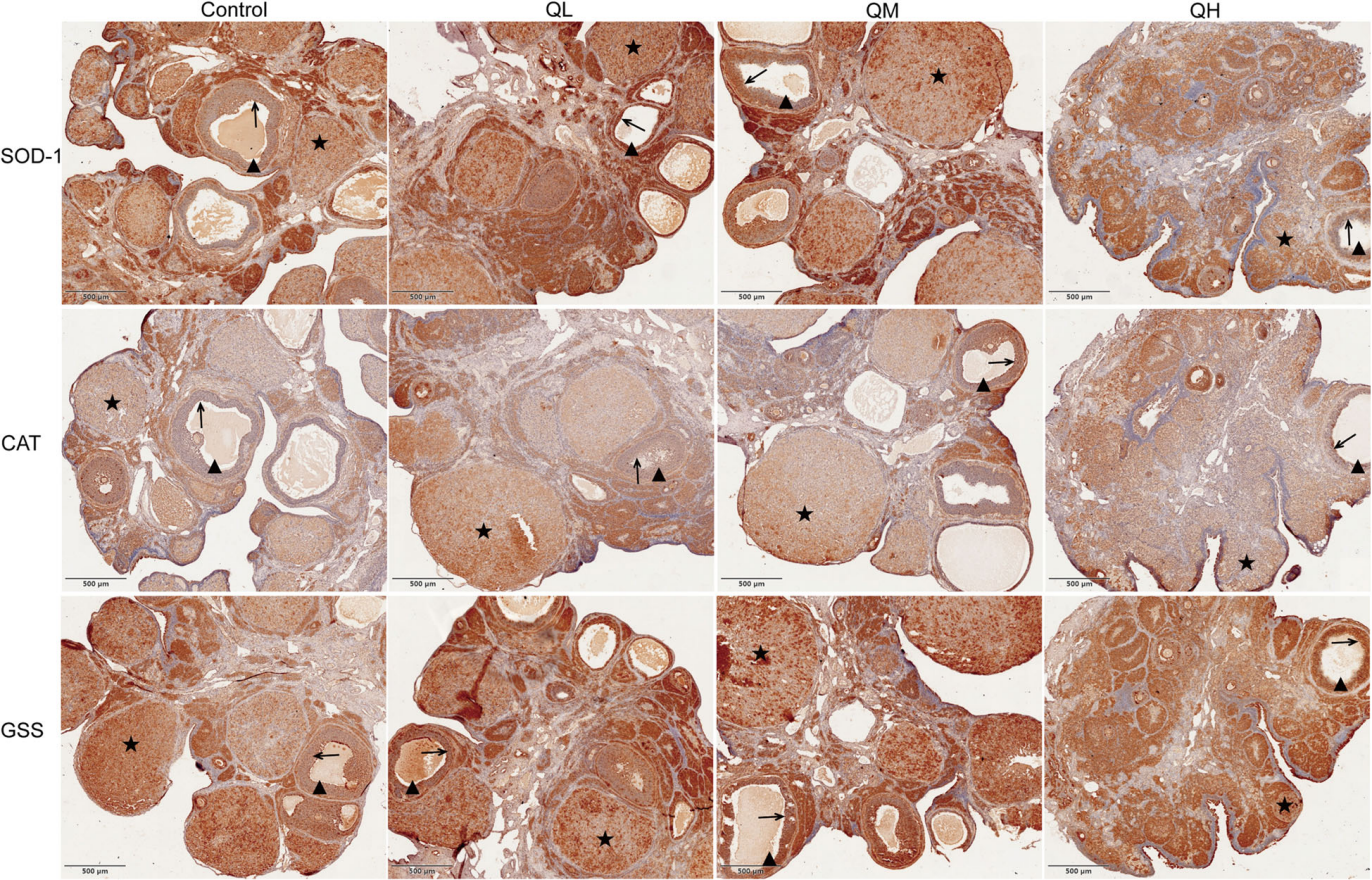
Protein expression levels and localizations of SOD-1, CAT and GSS in the ovary of menopausal rats. Scale bars, 500 μm. The expression levels of SOD-1, CAT, and GSS in the low-dose quercetin group were markedly higher than those in the control group. ★corpus luteum;▲growing follicles;→granulosa cell

The protein expression and localizations of SOD-1, CAT and GSS were further detected by immunohistochemistry (Fig. 5). The expression of SOD-1 and GSS in the ovary was primarily located in the granulosa cell of growing follicle and the luteal cell of corpus luteum. Compared with the control group, the expression levels of SOD-1, CAT, and GSS in rat ovary were higher in rats treated with low-dose and middle-dose quercetin. However, no significant differences were detected in the expression of these proteins between the high-dose quercetin group and the control group. Western blot and immunohistochemistry results indicated that quercetin within the range of doses used in this study could increase the protein expression of SOD-1, CAT and GSS in the menopausal rat ovary.

### Effect of quercetin on cell viability and estradiol production of rat ovarian granulosa cells

**Fig. 6:**
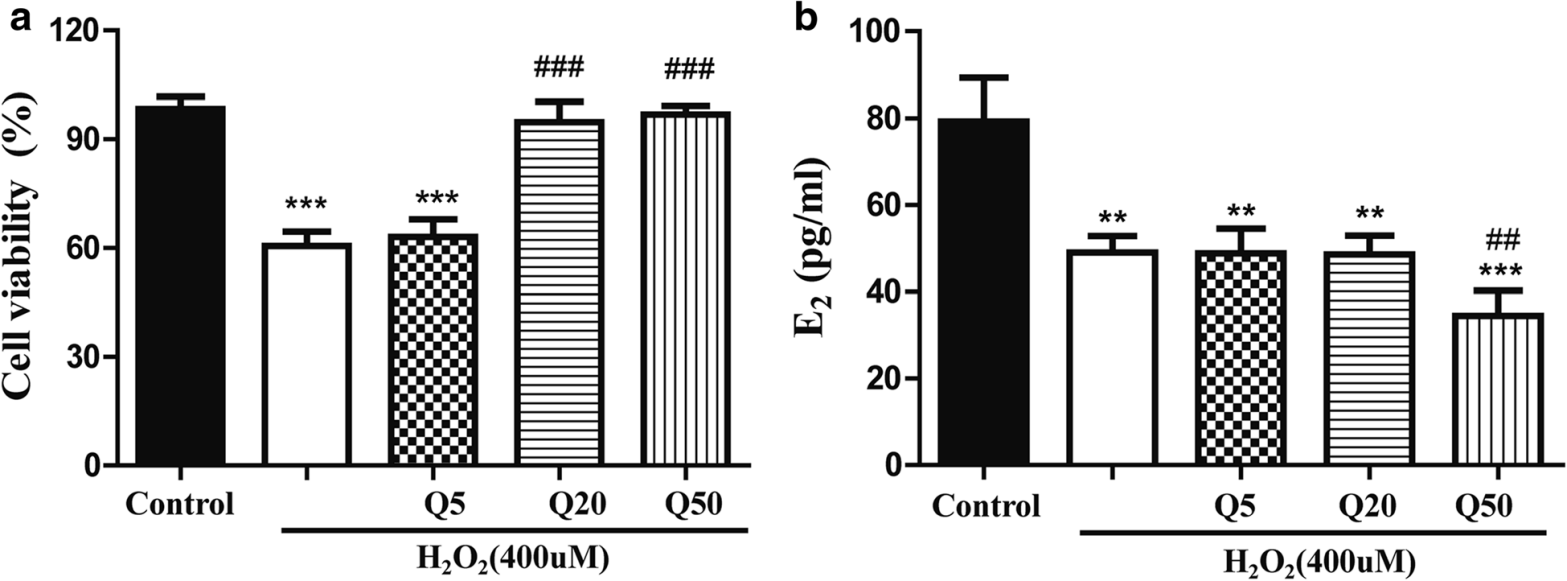
Effect of quercetin on cell viability and estradiol production of rat ovarian granulosa cells. Data are presented as the *Mean* ± *SD*. **a** Cell viability was detected via CCK-8 assay. Quercetin at 20 μM significantly rescued the decrease in cell viability caused by H_2_О_2_-induced oxidative stress, and 50 μM quercetin both rescued the H_2_O_2_-induced cell damage and ameliorated the H_2_O_2_-induced decrease in expression of oxidative stress-related proteins; **b** estradiol production was detected by radioimmunoassay. Quercetin did not rescue the _2_O_2_-induced decrease in estrogen. (comparing with control, ^**^P < 0.01, ^***^P < 0.001; comparing with H_2_О_2_, ^##^*P* < 0.01,^###^*P* < 0.001)

CCK-8 assay was used to detect the effect of quercetin on cell viability of cultured granulosa cells in vitro. Compared with the control group, cell viability decreased by approximately 40% in the H_2_O_2_ (400 μM) group (*P* < 0.001). However, when H_2_O_2_ was co-applied with various concentrations of quercetin (5 μM, 20 μM and 50 μM), we found that quercetin at 20 μM and 50 μM could rescue the H_2_O_2_-induced cell damage (*P* < 0.001, Fig. 6a). This result indicated that quercetin had protective effects on cultured granulosa cells in vitro.

Further, a radioimmunoassay was used to detect the effects of quercetin on estrogen production in cultured granulosa cells. The results showed that after H_2_O_2_ treatment, the secretion of estrogen decreased approximately 39% compared with the control group (*P* < 0.001, Fig. 6b). Unlike the cell viability results, quercetin did not rescue the H_2_O_2_-induced decrease in estrogen. This result indicated that the protective effect of quercetin on cultured granulosa cells was probably not related to estrogen secretion.

### Effects of quercetin on protein expression of SOD-1, CAT and GSS on rat ovarian granulosa cells

**Fig. 7:**
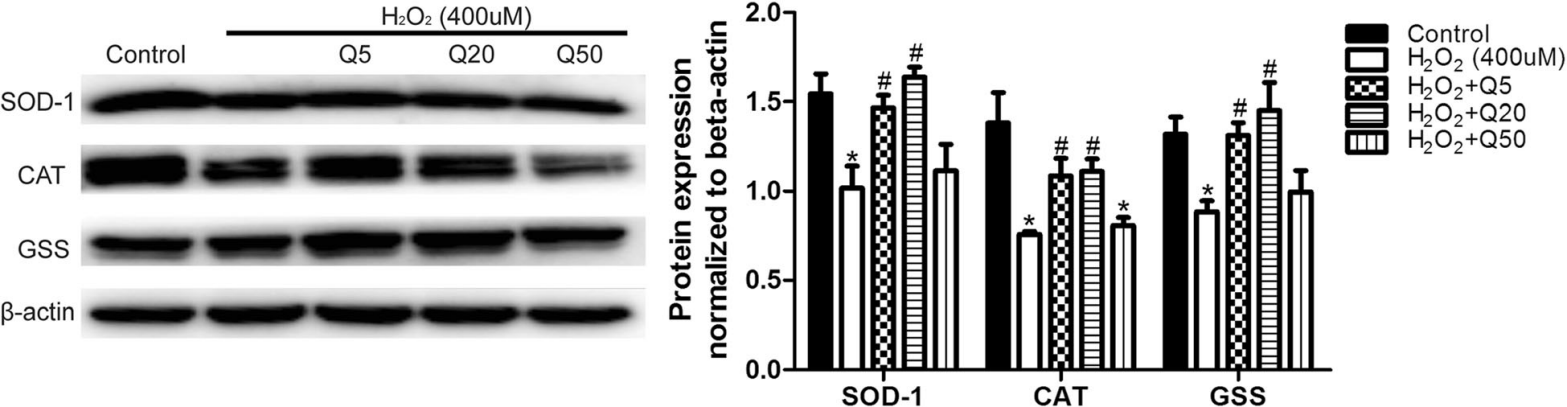
The protein expression of SOD-1, CAT and GSS in rat ovarian granulosa cells as detected by Western blot. Data are expressed as protein normalized to β-actin and are given as the *Mean* ± *SD*. The protein expression levels of SOD-1, CAT and GSS were reduced after H_2_O_2_ treatment, and co-incubation with quercetin and H_2_O_2_ showed higher expression levels of oxidative stress-related proteins compared with incubation with H_2_O_2_ alone. (comparing with control, ^*^P<0.05; comparing with H_2_O_2_, ^#^*P*<0.05)

The effects of quercetin on protein expression of SOD-1, CAT and GSS on rat ovarian granulosa cells were detected by Western blot. The results showed that the protein expression levels of SOD-1, CAT and GSS decreased after H_2_O_2_ treatment (*P* < 0.05), and quercetin could upregulate the expression of oxidative stress-related proteins when cells were co-incubated with H_2_O_2_ and quercetin (P < 0.05) (Fig. 7). This result indicated that the protective effect of quercetin on cultured ovarian granulosa cells may be related to the upregulation of oxidative stress-related protein expression.

## Discussion

In this study, we found that quercetin increased the antioxidant capacity of the ovary by upregulating expression of some oxidative stress-related genes both in vivo and in vitro.

The estrous cycle in female menopausal rats was determined before and after administration of quercetin. We found that most rats (28/30) had entered menopause with an irregular or prolonged estrous cycle. After administration of quercetin, no periodic change in the estrous cycle was observed in the quercetin groups compared with the control group. Consistent with the results of our study, a previous study showed no significant effect on the estrous cycle in early senescent rats (11 months old) after the administration of quercetin (50 mg/kg) for 4 months [16]. Other studies also find that estrous cycles become irregular or prolonged when rats begin menopause [18, 19]. Therefore, we speculate that quercetin has no significant effect on maintaining or reversing the estrous cycle of menopausal rats.

HE staining results showed that the ovaries of menopausal rats demonstrated obvious aging characteristics, including the exhaustion of the resting follicle reserve and a marked increase in the proportion of ovarian stroma. Additionally, we found that quercetin had no obvious effect on the morphology of the ovary in menopausal rats. This result is consistent with the results of Chen et al., who found that the numbers of healthy follicles and atretic follicles in quercetin-treated rats were not significantly different from those in the control group [16]. Combined with the results that quercetin had no effect on the weights of the ovary in menopausal rats, these data confirmed the conclusion that quercetin has no significant effect on the follicular reserve in menopausal rats.

At menopause, estrogen levels decline, whereas pituitary LH and FSH levels increase [20]. Kellis et al. reported that quercetin inhibits the aromatization of androstenedione to estrone and of testosterone to estradiol in human placental and ovarian microsomes [21]. However, Victor et al. found that quercetin increased protection against cadmium chloride (CdCl_2_)-induced imbalances in reproductive hormones (E_2_, P, FSH and LH) [13]. Our experimental results showed that the levels of E_2_, P, FSH, and LH in menopausal rats treated with quercetin were not significantly different from those of the control group. Although this result is inconsistent with the results of Victor et al., a possible reason might be the use of different animal models.

Oxidative stress is one of the primary causes of aging [22, 23]. Therefore, in this study, we first detected the serum levels of several antioxidant indices (T-AOC, SOD, GSH, GSH-PX, and GST), and the results showed no significant differences between the quercetin groups and control group. To investigate whether quercetin had an antioxidant effect on aging in the rat ovary, we further examined the expression of mRNA and proteins for the oxidative stress-related genes SOD-1, CAT, and GSS in the ovary. Compared with the control group, mRNA and protein expression levels of these oxidative stress-related genes were upregulated after treatment with low-dose quercetin. A recent report found that quercetin significantly increases the activity and level of SOD, CAT, GSH and GSH-PX in docetaxel-induced testicular damage [24], which is consistent with our findings. Of note, the administration of high-dose quercetin decreased the mRNA and protein expression levels of SOD-1, GSS and CAT in menopausal rats. We hypothesize that within a given dose range, quercetin could increase the antioxidant capacity and protect the body against the damage of free radicals; however, when the dose is too high, its antioxidant activity may be counteracted by other toxic or side effects of the drug.

To further confirm the in vivo result, primary ovarian granulosa cells were cultured in the present or absence of H_2_O_2_ (400 μM) and three concentrations of quercetin (5 μM, 20 μM, and 50 μM). We found that quercetin could rescue the H_2_O_2_-induced cell damage and ameliorate the H_2_O_2_-induced decreases in expression of oxidative stress-related proteins, indicating that the protective effect of quercetin on cultured ovarian granulosa cells may be related to the upregulation of expression of oxidative stress-related proteins. Recently, Gao reported that quercetin has a protective effect on hypoxia-induced damage to primary cultured retinal ganglion cells [25]. Another study showed the protective effects of quercetin against cell injury and inflammation in HUVECs [26]. Our results further confirm our hypothesis that quercetin at an appropriate dose can protect ovary function against damage caused by free radicals.

## Conclusions

**Fig. 8:**
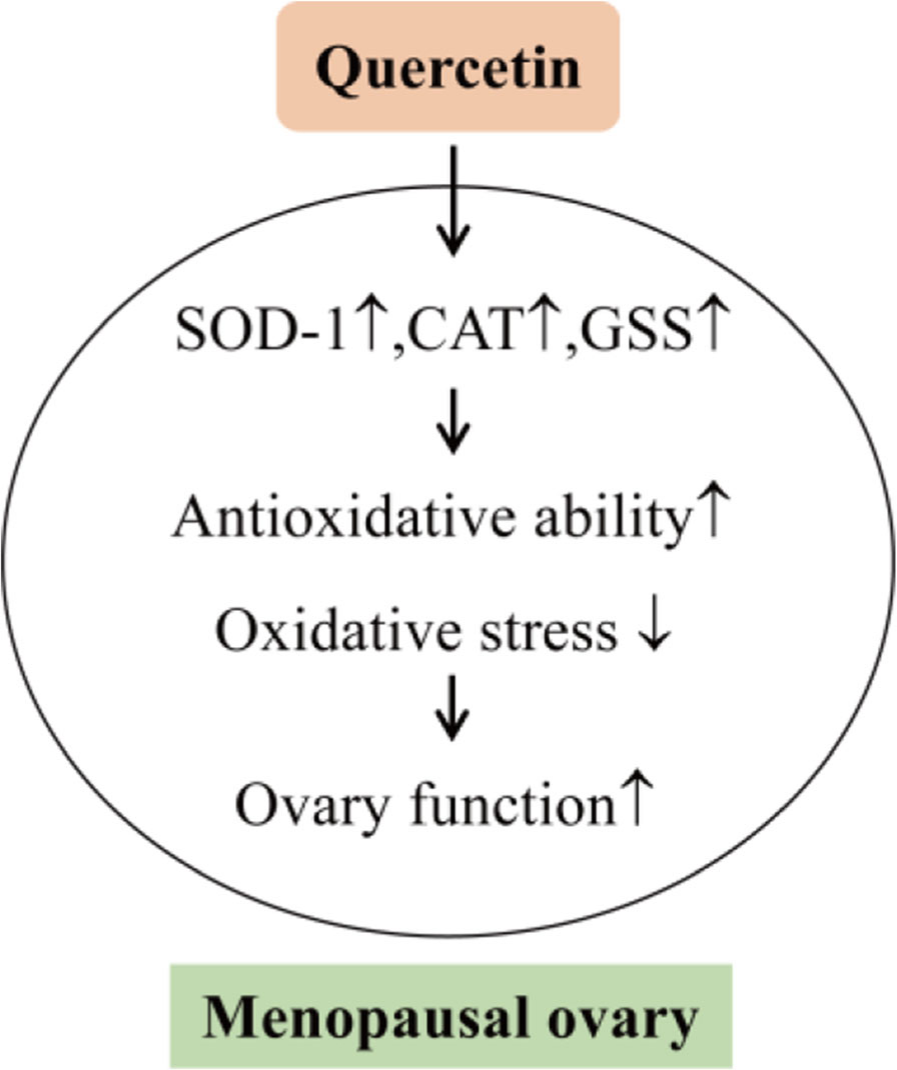
The hypnotized schematic diagram that effect of quercetin on menopausal ovary

A hypothesized schematic diagram based on these results is shown in Fig. 8. Although our results demonstrated that quercetin had no effects on ovarian morphology, hormone secretion, or the estrous cycle, low-dose quercetin caused the upregulation of oxidative stress-related genes in the ovary in menopausal rats, which could increase the antioxidant capacity of the ovary and delay ovarian aging. Further studies are required to determine whether low-dose quercetin can be used to prevent and treat menopausal complications by reducing oxidative stress in aging ovaries.

## Abbreviations

CAT: Catalase
E_2_: Estradiol
FSH: Follicle stimulating hormone
GSH: Glutathione
GSH-PX: Lutathione peroxidase
GSH-ST: Glutathione S-transferase
GSS: Glutathione synthetase
HE: Hematoxylin and eosin
HRP: Horseradish peroxidase
LH: Luteinizing hormone
P: Progesterone
PMSG: Pregnant mare serum gonadotropin
PVDF: Polyvinylidene difluoride
SOD: Superoxide dismutase
SOD-1: Superoxide dismutase-1
T-AOC: Total antioxidant capacity
